# Editorial: Avian Muscle Development and Growth Mechanisms: Association With Muscle Myopathies and Meat Quality

**DOI:** 10.3389/fphys.2020.601184

**Published:** 2020-11-04

**Authors:** Sandra G. Velleman, Massimiliano Petracci

**Affiliations:** ^1^Department of Animal Sciences, The Ohio State University, Wooster, OH, United States; ^2^Department of Agricultural and Food Sciences, Alma Mater Studiorum – University of Bologna, Bologna, Italy

**Keywords:** satellite cells, poultry, myopathies, meat, spaghetti meat, wooden breast

## Introduction

The Research Topic Avian Muscle Development and Growth Mechanisms: Association with Muscle Myopathies and Meat Quality is the first comprehensive treatise associating the basic biology of muscle growth with the incidence of breast muscle myopathies including the influence of genetics, effect on meat quality, and the development of new detection methods with more accuracy than standard phenotypic palpation. For this special Research Topic, a total of 20 manuscripts were accepted for publication: 13 original research contributions; 4 hypothesis and theory; and 3 reviews.

A couple of review papers explore different aspects of early and late muscle development which can be related to the occurrence of breast muscle myopathies in fast-growing broilers. Velleman summarizes recent findings on how the Wooden Breast myopathy is associated with fibrosis and re-assembling of extracellular matrix proteins with special emphasis on the fibril-forming collagens (namely type I and III) and its implications on breast muscle functionality and resulting meat quality in different fast-growing commercial hybrids with varying occurrence of phenotypically detectable wooden breast condition. Halevy presents a mini-review on how satellite cell proliferation and differentiation as well as prenatal muscle development and postnatal pectoral muscle growth, are dramatically affected by environmental conditions during chicken embryonic development and initial stages after hatching. This paper further reveals the extreme importance of duration and extent of the change in environmental thermal load and its timing during these stages in determining muscle development and growth in poultry, even in the occurrence of muscle myopathies. This topic is also explored by Oviedo-Rondón et al. who showed that metabolic disorders in avian species can start very early in life, and suboptimal incubation conditions may trigger some of the key alterations on muscle metabolism and be associated with the onset of myopathies. Zampiga et al. reviewed the current understanding of the breast myopathies and possible implications on the quality of turkey meat. Overall, it was clearly stated that the relevance and practical importance of emerging growth-related breast abnormalities in the turkey is limited if compared to the broiler chickens. Therefore, the occurrence of PSE-like condition is still the major issue for the turkey industry. On the same species, Horton et al. explored for the first time the role of a specific group of proteins involved in cellular apoptosis in the early stages of muscle growth and development. It was demonstrated that decreased expression of death-associated proteins significantly affects the transcriptome profile of satellite cells in turkey pectoral muscles, thus impairing its proliferation and differentiation ability.

Most of the original research covers several key topics in the growth of the breast and modifications in the growth process as it relates to incidence of breast muscle myopathies in broilers. Ferreira et al. describe alterations in the satellite cell population, macrophage population, and collagen content in wooden breast affected muscle. Satellite cells are responsible for post-hatch muscle growth and the repair of muscle with damage like that which occurs in fibrotic myopathies. In Wooden breast affected muscle, the number of proliferative satellite cells increased as did macrophage and collagen content. The wooden breast myopathy has been associated with hypoxia induced by rapid growth. Hosotani et al. describe how altered mitochondrial clearance induced by chronic hypoxia and injury to mitochondria are associated with the pathological features of wooden breast. Furthermore, Baldi et al. report that the elevated ultimate pH in wooden breast meat involves reduced glycolytic potential and impaired ATP energy generation which would impact the degree of muscle fiber contraction.

Thus, research contributions addressing how muscle myopathies affect the structure of the breast muscle which will ultimately alter meat quality were also included in this special Research Topic. In research addressing the cellular cytoarchitecture proteins desmin and vimentin by Soglia et al. affected breast muscles from wooden breast, white striping, and spaghetti meat myopathies were compared to normal breast muscle. It was found that wooden breast and white striping affected tissue differentially undergoing severe regeneration, and vimentin may be associated with the development of the spaghetti meat phenotype. With regard to sarcomere structure, the physiological properties of single muscle fibers from wooden breast affected muscle were compared to normal muscle by Liu et al.. They found that calcium sensitivity was significantly decreased in wooden breast muscle and wooden breast muscle fibers had 50% larger sarcomeric volume compared to normal muscle. However, the content of myosin and actin, and maximal shortening velocity were unchanged suggestive of the synthesis of new sarcomeres with unaltered kinetics.

To further a mechanistic understanding into the cause of breast muscle myopathies, cutting edge genomic and transcriptome research were included in this treatise. Bailey et al. present the first reported estimates of the genetic basis for breast muscle defects in broilers including deep pectoral myopathy, wooden breast, white striping, and spaghetti breast. They present evidence showing that breast muscle myopathies have a low genetic relationship with performance and that it is also important to understand non-genetic effects on myopathy incidence. Transcriptional profiling studies by Malila et al. on white striping severity in commercial broilers demonstrated intracellular ion imbalance, particularly calcium, oxidative stress, and impaired programmed cell death on the progression of white striping. Praud et al. report on molecular phenotyping of the white striping and wooden breast myopathies to potentially identify biomarkers for diagnostic purposes and to improve our understanding of the physiology of wooden breast and white striping. Wooden breast also has a differential occurrence between the sexes in broiler chickens. In male birds, the cranial region of the breast muscle tends to be more severely affected compared to females. Through RNA-Seq, Brothers et al. describe the increased expression of fat metabolism, oxidative stress response, antiangiogenesis, and connective tissue proliferation genes in male broilers. Thus, these data begin to demonstrate why male broilers are more susceptible to wooden breast.

A couple of the original research contributions propose advanced tools to detect wooden breast which poses significant meat quality issues and consumer complaints in the poultry industry. In the first, Morey et al. investigated the potential use of bioelectrical impedance analysis. Very promising results were obtained when normal and wooden breast samples were compared, while it was not possible to differentiate between varying severity levels of wooden breast myopathy. In addition, the accidental outcome on the interference of spaghetti breast meat opens new research areas to investigate the ability of this analytical approach to also identify this condition. On the other hand, Phillips et al. explored for the first time the application of machine learning tools for extracting critical information contained in gene expression data obtained from pectoralis major muscle and liver of chickens exhibiting wooden breast condition and those that were normal. This innovative approach allows to establish that evolution of wooden breast involves many molecules and pathways thus suggesting that the etiology of this condition is associated not only to muscle activity but altered systemic pathology. These findings were also indirectly supported by the study of Lake et al. which reveals that the influence of wooden breast is not limited to the pectoral muscles. Indeed, it was evidenced that wooden breast is coupled with blood gas disturbances characterized primarily by the accumulation of carbon dioxide and acidification of venous blood which can be likely associated with impairment of pulmonary gas exchange. Consequently, there is good evidence to suggest that birds affected by wooden breast condition can have an increased metabolic rate that may also be poorly compensated due to cardiovascular deficiencies such as insufficient venous return or respiratory deficiency. In addition, Mallmann et al. speculated that, as in mammals, deviations in bone metabolism may be associated with growth-related abnormalities occurring in modern fast-growing broilers. A clear association emerged between distinctive histological alterations (abundant infiltration of adipose tissue, muscle fibers degeneration and necrosis, infiltration of heterophils and mononuclear cells, connective tissue proliferation, and vasculitis) and abundant infiltration of adipose tissue bone marrow in broilers affected by wooden breast condition. Indeed, Lake and Abasht suggest a new hypothesis on the etiology of wooden breast that involves impairment of lipid and glucose metabolism in consequence of the proposed similarities between wooden breast and type 2 diabetes regardless of its phenotypic dissimilarities. They hypothesize that these dissimilarities are due to different glucose transport metabolism between avian and mammalian skeletal muscle, and that the wooden breast phenotype most closely resembles complications of diabetes in smooth and cardiac muscle of mammalian species. Finally, Oviedo-Rondón and Córdova-Noboa summarizes current knowledge on the potential of guanidino acetic acid dietary use to mitigate growth-related breast abnormalities with emphasis on the wooden breast myopathy and the possible use of plasma creatine and related blood enzymes as markers for early detection of this condition in live birds.

## Author Contributions

The contributions are all original to the Research Topic. All authors contributed to the article and approved the submitted version.

## Conflict of Interest

The authors declare that the research was conducted in the absence of any commercial or financial relationships that could be construed as a potential conflict of interest.

